# Inoculation with Indole-3-Acetic Acid-Producing Rhizospheric *Rhodobacter sphaeroides* KE149 Augments Growth of Adzuki Bean Plants Under Water Stress

**DOI:** 10.4014/jmb.1911.11063

**Published:** 2020-02-10

**Authors:** Sang-Mo Kang, Arjun Adhikari, Ko-Eun Lee, Muhammad Aaqil Khan, Abdul Latif Khan, Raheem Shahzad, Sanjeev Kumar Dhungana, In-Jung Lee

**Affiliations:** 1 School of Applied Biosciences, Kyungpook National University, Daegu 4566, Republic of Korea; 2 Natural and Medical Science Research Center, University of Nizwa, Nizwa 616, Oman; 3 Department of Biology, College of Science, Imam Abdulrahman Bin Faisal University, P.O. Box 1982, Damam 1441, Saudi Arabia; 4 Basic and Applied Scientific Research Center, Imam Abdulrahman Bin Faisal University, P.O. Box 1982, Damam 311, Saudi Arabia; 5 Department of Southern Area Crop Science, National Institute of Crop Science Rural Development Administration, Miryang 0424, Republic of Korea

**Keywords:** Abiotic stress, drought, flood, phytohormones, *Rhodobacter sphaeroides* KE149

## Abstract

The use of plant growth-promoting rhizobacteria is economically viable and environmentally safe for mitigating various plant stresses. Abiotic stresses such as flood and drought are a serious threat to modern agriculture. In the present study, the indole-3-acetic acid-producing rhizobacterium *R. sphaeroides* KE149 was selected, and its effects on the growth of adzuki bean plants under flood stress (FS) and drought stress (DS) were investigated. IAA quantification of bacterial pure culture revealed that KE149 produced a significant amount of IAA. Moreover, KE149 inoculation notably decreased stress-responsive endogenous abscisic acid and jasmonic acid and increased salicylic acid in plants under DS and FS. KE149 inoculation also increased proline under DS and methionine under FS. In addition, KE149 inoculation significantly increased the levels of calcium (Ca), magnesium (Mg), and potassium (K) while lowering the sodium (Na) content in the plant shoot under stress. KE149-treated plants had markedly greater root length, shoot length, stem diameter, biomass, and higher chlorophyll content under both normal and stressed conditions. These results suggest that KE149 could be an efficient biofertilizer for mitigating water stress.

## Introduction

The adzuki bean plant is a leguminous plant consumed as folk medicine and is one of the important crops especially in countries such as China, Korea, and Japan [1]. It has rich antioxidant properties and medicinal importance [2]. However, studies on adzuki bean cultivation are limited [3]. Abiotic factors such as flood, drought, salinity, extreme temperature, and nutrient unavailability are considered as the major causes for a reduction of almost 50% of the potential crop yield [4]. Drought stress has led to crop production damage (> 7%), which is 8–11% greater in developed countries compared with developing countries globally [5]. Despite various scientific approaches, the amelioration of drought stress and flood stress is still challenging for quality crop production. Accordingly, a better understanding of the physiology beyond flood stress and drought stress and its biological remediation could broaden the scope of research.

In order to adapt to stress, plants attempt to morphologically change their intercellular formation through the induction of several signaling regulators such as plant hormones and ROS [6]. Among the various phytohormones, indole-3-acetic acid (IAA), salicylic acid (SA), abscisic acid (ABA), and jasmonic acid (JA) play an important role in signaling and cross-communication. Under flood stress, plants are unable to absorb oxygen for normal physiological activities because they cannot generate glucose, causing several metabolic problems. During drought, plants tend to accumulate ABA, and auxin levels are usually decreased [7].

A mechanism that has been identified in rice under drought is that JA can modulate root hydraulic conductivity to enhance drought tolerance. Specifically, the rice bHLH protein (OsbHLH148) interacts with O_S_JAZ1 (jasmonic acid-regulated gene) and activates OsDREB1 (a protein for drought tolerance), enhancing drought stress tolerance. In addition, the biosynthesis and transport of ABA are mediated by ABA signaling receptors (2C-type protein phosphatases), which transport ions to the stomata, resulting in the influx of CO_2_ and efflux of H_2_O and leading to stomatal closure [8]. SA biosynthesis requires the primary metabolite chorismate, which involves isochorismate synthase (ICS) and phenylalanine ammonia lyase (PAL) [9]. Moreover, under drought, AUX/IAA is conjugated to amino acids, which is catalyzed by GH3 proteins. IAA/AUX is localized in cells and degraded when it interacts with auxin-signaling F-Box protein. After degradation, auxin response factors are released, which mediate the transcription of auxin-responsive genes [8].

In recent years, plant growth-promoting rhizobacteria (PGPR) have been reported to mitigate plant stress and promote growth through phytohormone modulation, antioxidant and amino acid regulation, and nutrient assimilation. These microorganisms colonize the root rhizosphere and acquire several micronutrients and macronutrients such as calcium (Ca), magnesium (Mg), potassium (K), and phosphorus (P) from the soil. These nutrients play a key role in regulating the amino acid pathway. Amino acids are considered as precursors for the defense system in plants, and they play an important role in the cross talk between SA and JA, affecting the plant-microbe interaction [10, 11]. Amino acids are osmotically active substances that contribute to osmotic pressure adjustments during water stress. Amino acids maintain the negative osmotic potential of cells during drought stress, thus keeping the cell wall rigid and preventing cells from shrinking due to dehydration [12]. Under flood stress, methionine has been reported to act as a precursor in the synthesis of ethylene, which is produced through 1-aminocyclopropane-1-carboxylic acid (ACC) oxidase and ACC synthase. During ethylene biosynthesis, ACC is converted to ethylene, and oxygen is vital for the production of ethylene. The absence of oxygen in the root zone during flood may disrupt plant growth [7]. The application of PGPR could regulate plant metabolism by enhancing methionine production and promoting plant growth. These effects have been reported for several microorganisms such as *Pseudomonas putida* [13], *Promicromonospora* sp. SE188, *Burkholderia cepacia* *SE4*, and *Acinetobacter calcoaceticus* SE370 [14].

In our previous study, *Rhodobacter sphaeroides* was found to promote the growth of cucumber plants by producing IAA [15]. Our current analysis further revealed the innate ability of the *R. sphaeroides* strain to produce IAA. [16]. IAA producing bacteria effectively protect plants against various biotic and abiotic stresses and can assist plant growth [17]. Rhizobacteria use tryptophan in root exudates and convert them into IAA, which is utilized by the plant roots and hence activate the plants auxin signaling pathway, which ultimately led to proliferation and growth promotion of plant cells [18]. Therefore, we hypothesized that the application of IAA-synthesizing *R. sphaeroides* could confer resistance against flood stress and drought stress in soybean plants.

## Materials and Methods 

### Bacterial Inoculum Preparation

The bacterial strain was grown in 250 ml of LB broth (tryptone 10 g, yeast extract 5 g, NaCl 10 g, pH 7.0 ± 0.2; autoclaved for 15 min at 121°C) for 5 days and centrifuged at 5,000 ×*g* for 15 min to separate the culture broth and bacterial cells. The cells were separated, diluted with sterilized distilled water and used for inoculation in plants.

### IAA Detection and Quantification

The method described by Lee *et al*. [19] was followed to detect and quantify the IAA production in KE149 culture. The details are mentioned in supplementary file.

### Extraction and Quantification of ABA in Bacterial Pure Culture 

ABA was extracted and quantified using the method described by Shahzad *et al*. [20]. In brief, *R. sphaeroides* was grown in LB media (tryptone 10 g, yeast extract 5 g, NaCl 10 g, pH 7.0 ± 0.2; autoclaved for 15 min at 121°C) for 3 and 5 days. Then, 25 ml of broth was taken, and the pH was adjusted to 2.5. The extract was obtained using ethyl acetate. The pure culture filtrate in which *R. sphaeroides* KE149 was grown was supplemented with [(±)-3,5,5,7,7,7-d^6^]-ABA as an internal standard. Polyvinylpolypyrrolidone was added, and the mixture was stirred at 120 rpm for 40 min, followed by filtration; the pH was adjusted to 3.5, and the mixture was partitioned by EtOAc. The extracts were left to evaporate, and ABA was obtained using diethyl ether/methanol (3:2, v/v) and dichloromethane. The purified ABA extracts were dried, methylated, and analyzed by GC-MS/SIM (6890N Network GC System and 5973 Network Mass Selective Detector; Agilent Technologies, USA). The Lab-Base (ThermoQuset, UK) data system software was used to monitor the signal ions at *m/z* 162 and 190 for Me-ABA and *m/z* 166 and 194 for Me-[^2^H_6_]-ABA. The experiment was repeated three times.

### Plant Growth Conditions

The experiment was conducted in a greenhouse at Kyungpook National University, Daegu, South Korea. The temperature maintained in the greenhouse was not less than 25°C during the night and not above 35°C during the day. The crops were grown under natural light without any external supplementation from June to July. Adzuki bean (*Vigna angularis var. nipponensis cv. Arari*) seeds were carefully washed with deionized double-distilled water and treated with 70% ethanol for 30 sec followed by 2.5% sodium hypochlorite for 20 min and then washed with deionized double-distilled water three times. The sterilized seeds were then sowed in a plastic tray with autoclaved soil consisting of peat moss (13–18%), perlite (11%), zeolite (6–8%), and coco-peat (63–68%), which contained the macronutrients NO_3_^-^ (~0.205 mg/g), P_2_O_5_ (~0.35 mg/g), NH_4_^+^ (~0.09 mg/g), and K_2_O (~0.1 mg/g). The same soil was used for the entire experiment. Two weeks later, uniform and equal sized seedlings were selected and transplanted to a crate (44 cm × 24 cm × 19 cm) filled with soil up to 12 cm. After one week of transplantation, the plants were treated with 50 ml of cell suspension (1 × 10^8^ Colony-forming unit (CFU)/ml) for 7 days. The plants were normally irrigated during the treatment period. After 7 days of treatment with the bacterial culture, the plants were exposed to natural drought conditions and excess water stress. The natural drought stress conditions (-1.2 MPa) under which plants were exposed to water-deficient conditions for 10 days and excess water up to 6 cm above the surface soil was supplied in the case of flood stress for 10 days. The treatment groups were designated as follows: no stress (NS) with or without KE149 inoculation, drought stress (DS) with or without KE149 inoculation, and flood stress (FS) with or without KE149 inoculation. The present study was conducted in completely randomized design comprised of six replication. Each replicate consisted of eight plants grown in a crate. Irrigation in the entire experiment was performed using sterilized distilled water. After 10 days of stress, plant growth attributes such as shoot length, root length, fresh weight, stem diameter, and chlorophyll content were measured from randomly selected 3 replications per treatment. The remaining samples from other three replicates were uprooted, immediately dipped in liquid nitrogen, freeze-dried (IlShinBioBase Lyophilizer, PUTFDorea) ground, and kept at −80°C for biochemical analysis.

### Extraction and Quantification of Hormones in Plant Shoot

**Abscisic acid (ABA).** The method described by Khan *et al*. [21]was used to quantify ABA. In brief, 0.5 g of freeze-dried ground samples were extracted with 95% isopropanol and 5% acetic acid. The extract was filtered, and 0.5 ml of the [(±)-3,5,5,7,7,7-d^6^]-ABA internal standard was added. The extract was washed with 1 N NaOH, and the pH was adjusted to 12.5. Chlorophyll was removed by sequential extractions with CH_2_Cl_2_, followed by the addition of EtOAc. The supernatant was collected, concentrated, and washed with phosphate buffer (pH 8.0), and polyvinylpolypyrrolidone was added. The mixture was then stirred at 120 rpm for 40 min, followed by filtration; the pH was adjusted to 3.5, and the mixture was partitioned by EtOAc. Subsequently, extraction and quantification were performed as described in section 2.3.

**Salicylic acid (SA).** SA was extracted and quantified using the method of Seskar *et al*. [23]. The lyophilized samples were extracted with 100% methanol, vacuum-dried, and suspended in trichloroacetic acid (5%). The supernatant was separated using cyclopentane, ethyl acetate, and isopropanol, followed by the drying of the organic layer with nitrogen gas and suspension in 70% methanol. Analysis was performed on a HPLC system (Shimadzu RF-10AXL fluorescence detector). The excitation and emission peaks were at 305 nm and 365 nm, respectively. The flow rate was 1.0 ml/min. To quantify SA, standard peak values were used.

**Jasmonic acid (JA).** The method described by Adhikari *et al*. [24] was used to determine the JA content of the plant. In brief, the freeze-dried ground sample was suspended in a mixture of acetone and 50 mM citric acid. The [9,10-2H_2_] JA standard (50 ng) was added. The organic solvent was allowed to evaporate overnight to avoid the loss of volatile fatty acids. The solution was then filtered and extracted with diethyl ether. The extract was passed through a solid-phase extraction cartridge (500 mg of sorbent, aminopropyl). JA was recovered from the cartridge with diethyl ether and acetic acid (98:2 v/v), and the solvents were evaporated. The residue was esterified by the addition of diazomethane and dichloromethane. The obtained extract was analyzed by GC-MS (6890N Network GC System and 5973 Network Mass Selective Detector; Agilent Technologies, USA). Standard peak areas were used to estimate the JA content.

### Assay of Chlorophyll Content

The method described by Butts *et al*. [26] was used to determine the chlorophyll content of the leaves. Measurements were performed using the CCM-300 Chlorophyll Content Meter (Opti-Sciences, USA). The emission ratio of red fluorescence at 700 nm to far-red fluorescence at (735 nm) was determined.

### Amino Acid Analysis

The amino acid content was analyzed using the method described by Kang *et al*. [14]. In brief, 0.05 g of freeze-dried powdered plant sample were kept in a 4 ml vile and was hydrolyzed with 1 ml of 6N HCl at 110°C after charging with Nitrogen for 24 h. The cap of vile is opened after 24 h and temperature is reduced to 80°C and allowed to dry. After hydrolysis, the extract obtained was placed in an automatic amino acid analyzer (L-8900; Hitachi, Japan). A standard peak value was used to quantify the amino acid content. During the acid hydrolysis some amino acids like cysteine, cysteine are degraded and threonine, serine, isoleucine, valine results could not be liberated completely. Therefore, statistical analysis were performed on those amino acid of significant and authentic values.

### Elemental Analysis

The method described by Bilal *et al*. [25] was used to determine the elemental content in the plants. In brief, 0.5 g of the finely powdered freeze-dried sample was digested with HNO_3_, followed by heating, evaporation, and filtration. The obtained solution was subjected to inductively coupled plasma emission spectroscopy (Optima 7900DV, PerkinElmer, USA) for quantification.

### DPPH and Polyphenol Analysis

The method described by Adhikari *et al*. [27] was used with slight modifications to measure the DPPH radical scavenging activity. First, 0.05 g of the freeze-dried ground sample was shaken for 6 h with 100% methanol and centrifuged. The supernatant was collected, and 0.1% of freshly prepared DPPH solution was added to an equal volume of the sample and kept in the dark for 30 min at room temperature. The absorbance was measured using a spectrophotometer (Multiskan GO; Thermo Fischer Scientific, Finland) at 517 nm. Quantification was performed using the formula: DPPH radical scavenging ability (%) = [1 − (A1 − A2) / A3)] × 100, where A1 is the absorbance of an equal volume of the sample extract and DPPH, A2 is the absorbance of the sample extract and methanol, and A3 is the absorbance of DPPH and methanol.

The protocol described by Adhikari *et al*. [27] was used to calculate the total polyphenol content. In brief, 0.05 g of the freeze-dried ground sample was diluted with absolute methanol and centrifuged, and the supernatant was mixed with 1 ml of Na2CO3 (2%). Then, 50 μl of Folin-Ciocalteu reagent (1 N) was added, followed by incubation for 30 min in the dark at room temperature. The absorbance was measured at 750 nm using a spectrophotometer (Multiskan GO; Thermo Fischer Scientific). Galic acid was used as a standard.

### Statistical Analysis

The present study was conducted in a completely randomized design (CRD) comprised of eight replications. Each replicate consisted of eight plants grown in a crate. However, we used only six replications on our analysis. Plant growth attributes such as shoot length, root length, fresh weight, stem diameter, and chlorophyll content were measured in randomly selected three replications. The remaining plants were uprooted for biochemical analyses like ABA, SA, JA, Amino acid, Mineral elements, DPPH, and polyphenol were conducted in randomly selected three replications. Each replication represented an average value of randomly selected six plants grown in each crate. Statistical analysis was performed with SAS 9.4 software (SAS Institute, USA). The mean values among treatments were separated using Duncan’s multiple range test at *p* < 0.05. The graphical sketches were drawn using version 6.0 GraphPad Prism (USA). All experiments were performed in triplicate.

## Results

### Indole-3-Acetic Acid Producing Ability of *R. sphaeroides* KE149

**Fig. 1:**
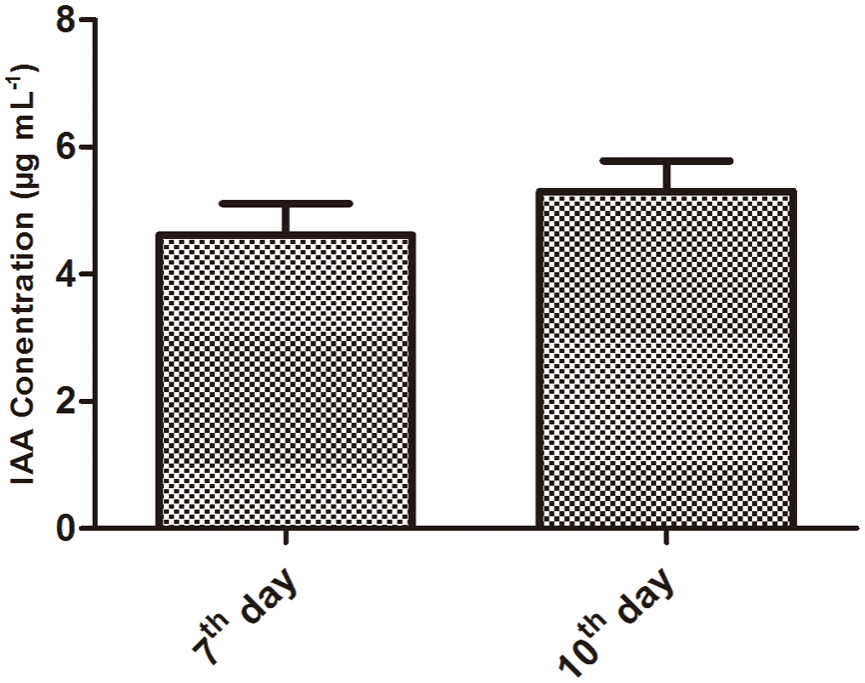
Quantification of Indole-3-acetic acid in *Rhodobacter sphaeroides* KE149 culture media.

IAA production ability of KE149 is detected through salkowski reagent test. The change in red color revealed the strain ability to produce IAA. Moreover, IAA quantification results showed that KE149 produced (4.6 ± 0.49 μgml^−^1) of IAA on the 7th day and (5.3±0.48 μgml^−^1) in 10th day after inoculation in the Luria Bertani (LB) culture broth (Fig. 1).

### Effect of *R. sphaeroides* KE149 on the Chlorophyll Content of Plants

**Fig. 2:**
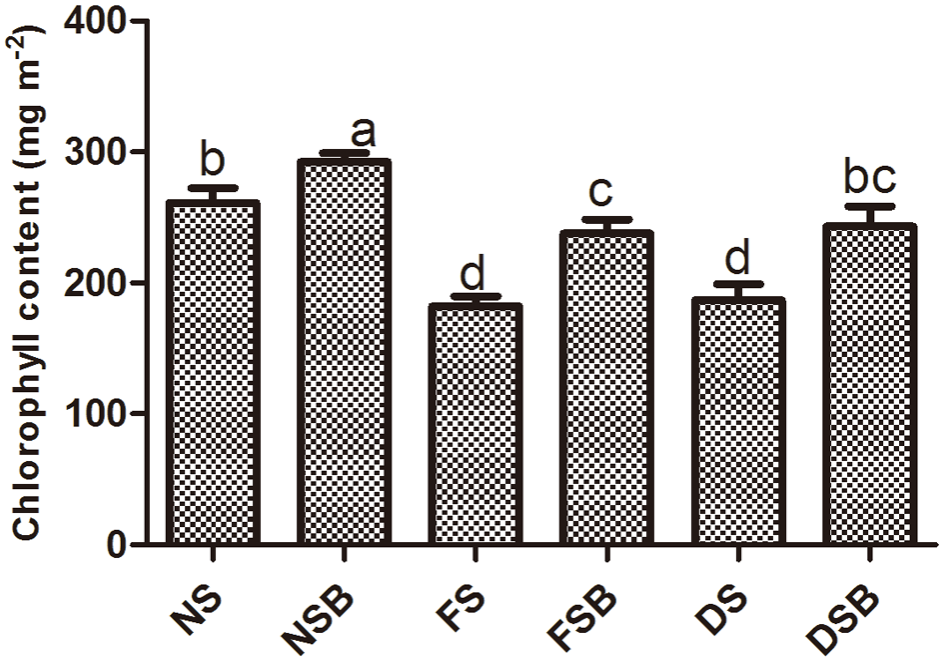
Effect of *R. sphaeroides* KE149 inoculation on the chlorophyll content of adzuki bean plants grown under water stress.

KE149 application increased the chlorophyll content of plants under both stressed and unstressed conditions. Overall, the chlorophyll content of bacterial inoculated plants was significantly higher under normal conditions. Under flood stress and drought stress, the chlorophyll content was significantly reduced; however, following bacterial inoculation, its level was significantly enhanced. Under normal conditions, the chlorophyll content was 30% higher in bacterial inoculated plants compared with non-inoculated plants. A similar pattern was observed under both drought stress and flood stress, where KE149 inoculation significantly increased the chlorophyll content by 30% compared with the content of non-inoculated plants (Fig. 2).

### Effect of *R. sphaeroides* KE149 on the Growth Attributes of Plants 

KE149 inoculation significantly increased plant growth attributes under both normal and stressed conditions. Under normal conditions, KE149-inoculated plants showed increases of 21.9%, 36.4%, 21.9%, 138.2%, and 17.5%in the shoot length, root length, shoot weight, root weight, and root diameter, respectively. Under flood stress, KE149 inoculation increased the shoot length by 26.1%, root length by 12%, shoot weight by 36.7%, root weight by 60%, and stem diameter by 6.6%. A similar pattern was observed under drought stress, where KE149 inoculation increased the shoot length by 31.3%, root length by 19.4%, shoot weight by 50%, root weight by 69%, and stem diameter by 11.4% (Table 1).

### Effect of *R. sphaeroides* KE149 on Plant Hormones

**Fig. 3:**
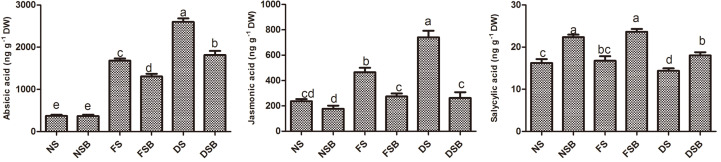
Endogenous levels of the phytohormones abscisic acid (ABA), jasmonic acid (JA), and salicylic acid (SA) following inoculation with *R. sphaeroides* KE149 in the root zone of soybean plants under flood stress and drought stress.

Overall, bacterial inoculation reduced ABA and JA levels and increased SA levels compared with the levels in non-inoculated plants. The results showed that under flood stress, ABA was significantly decreased by 22.12% in KE149-inoculated plants compared with non-inoculated plants. A similar pattern was observed under drought stress, where KE149 inoculation significantly reduced ABA by 30.23%. Similarly, KE149 inoculation significantly reduced JA by 41% and 64.64% under flood stress and drought stress, respectively. However, significantly higher (*p* < 0.05) levels of SA (41% under flood stress and 64.6% under drought stress) were observed in KE149-inoculated plants compared with non-inoculated plants. Under normal conditions, KE149 inoculation decreased JA and increased SA by 25%; however, there was no significant difference in ABA (Fig. 3).

### Effect of Microbial Inoculation on Mineral Nutrient Content in the Adzuki Bean Plant Shoot

**Fig. 4:**
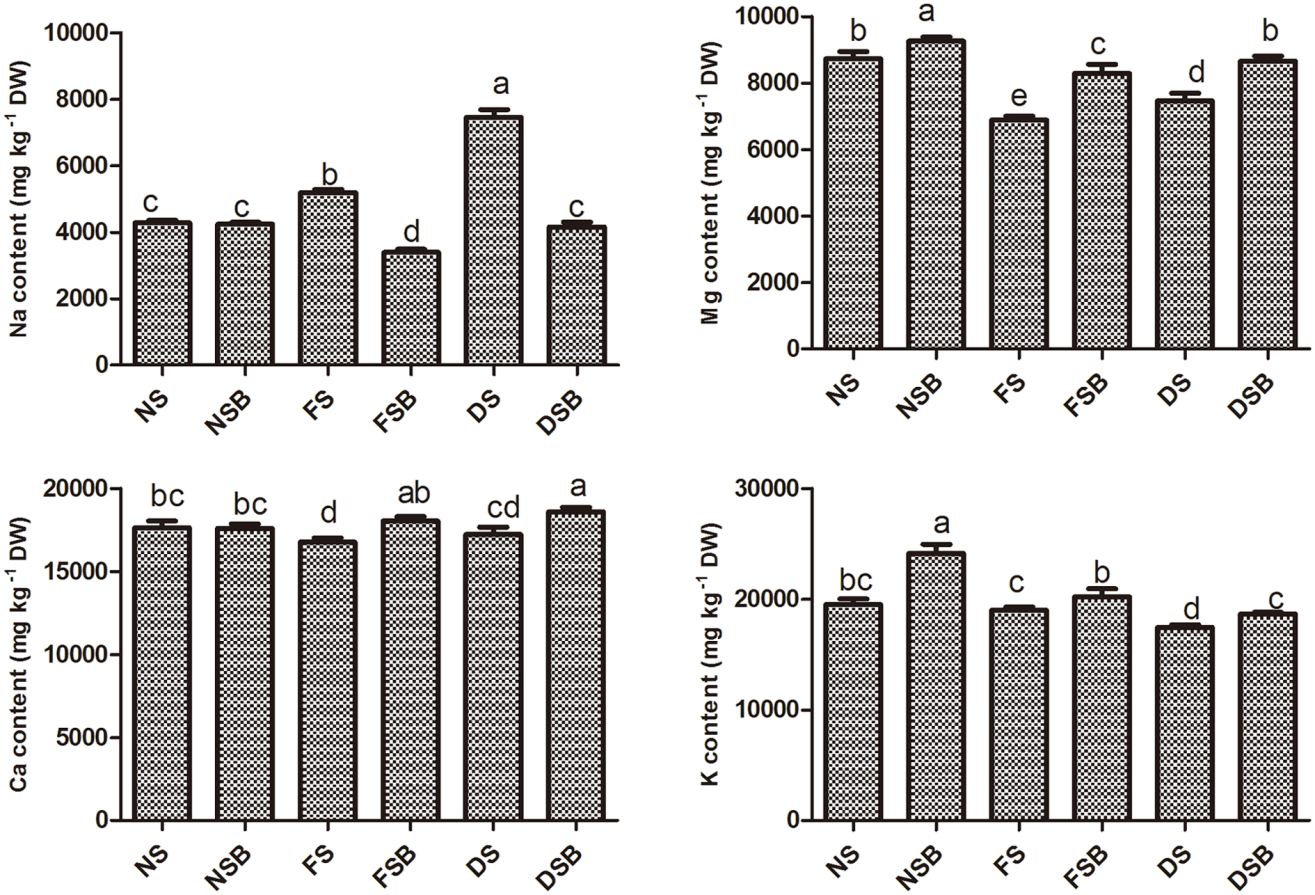
Elemental analysis of calcium (Ca), magnesium (Mg), potassium (K), and sodium (Na) in soybean plants following inoculation with *R. sphaeroides* KE149 under flood stress and drought stress.

Our study revealed that *R. sphaeroides* KE149 application significantly increased the Ca, K, and Mg content under both drought stress and flood stress. Under flood stress, KE149 inoculation increased the Ca content by 7.5%, K content by 6.4%, and Mg content by 20.5% compared with the content in non-inoculated plants. Under drought stress, KE149 inoculation increased the Mg content by 16%, Ca content by 8%, and K content by 7% compared with the content in non-inoculated plants. However, the sodium (Na) content was significantly reduced by 34.5%under flood stress and by 44.19% under drought stress in KE149-inoculated plants. Under normal conditions, there was no significant difference in the Na and Ca content; however, K (23.68%) and Mg (6%) were considerably higher in KE149-inoculated plants (Fig. 4). Overall, Na and Ca were significantly higher under drought stress and drought stress with bacterial inoculation, respectively, and Mg and K were significantly higher under no stress with bacterial inoculation.

### Analysis of Amino Acid Content

**Fig. 5:**
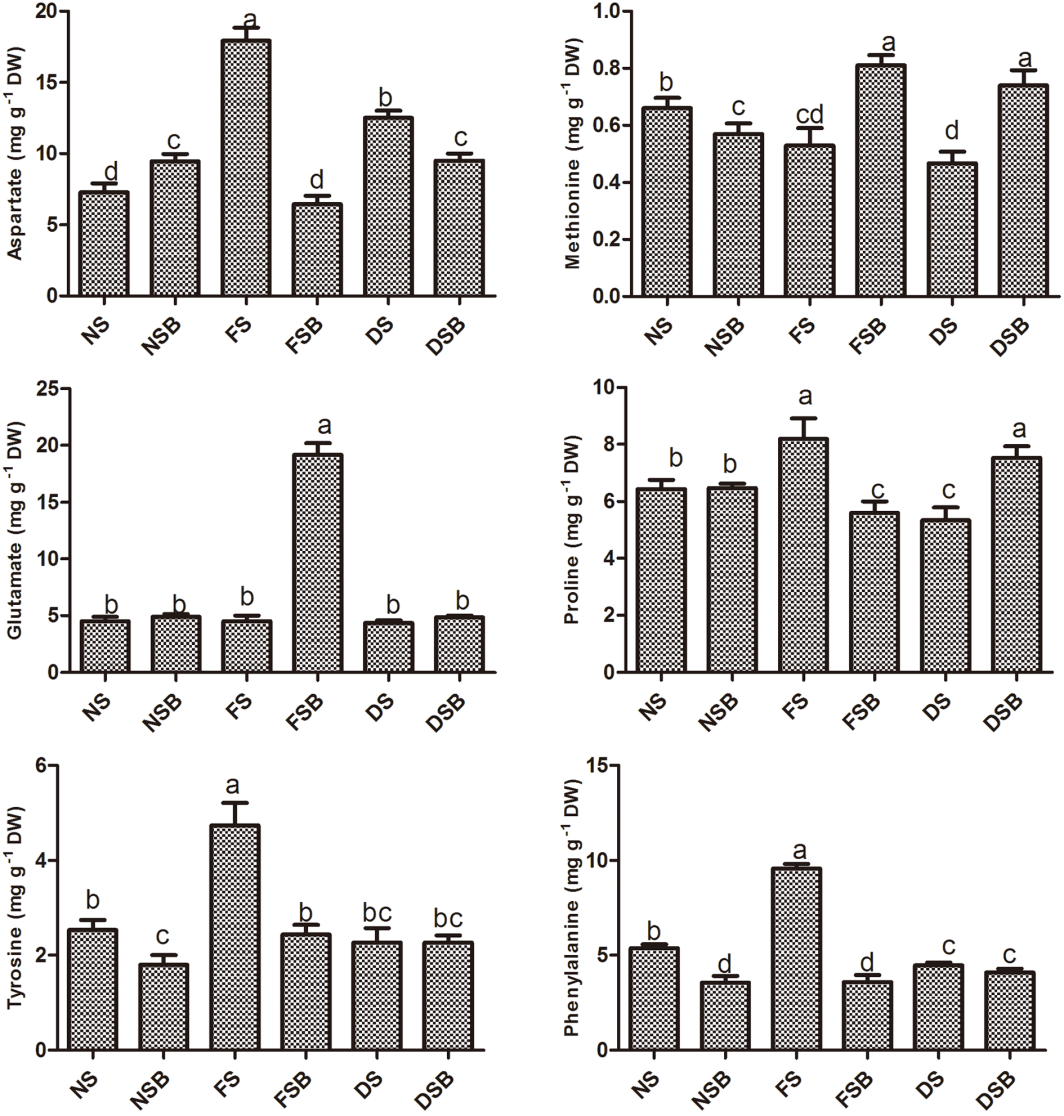
Quantification of amino acid content in adzuki bean plants grown under flood stress and drought stress with or without *R. sphaeroides* KE149 inoculation.

In our study, except methionine and glutamate, the amino acids aspartate, tyrosine, phenylalanine, and proline were significantly reduced under flood stress in KE149-inoculated plants compared with non-inoculated plants. However, all these amino acids except aspartate were significantly increased under drought stress in KE149-inoculated plants. Moreover, under no stress, bacterial inoculation increased aspartate and proline and reduced methionine, tyrosine, and phenylalanine in the plant shoot (Fig. 5).

### Polyphenol and 2,2-Diphenyl-1-Picrylhydrazyl (DPPH) Analysis

**Fig. 6:**
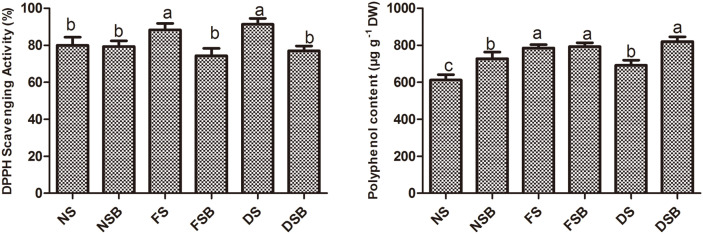
DPPH radical scavenging activity and polyphenol content in adzuki bean plants grown under flood stress and drought stress with or without *R. sphaeroides* KE149 inoculation.

Overall, the DPPH activity in bacterial inoculated plants was reduced under both flood stress and drought stress. In addition, the polyphenol content was increased under normal conditions and drought stress. KE149-inoculated plants exhibited significantly lower DPPH radical scavenging activity (< 15%) under both flood stress and drought stress. However, under normal conditions, bacterial inoculation did not result in significant differences in DPPH radical scavenging activity. Although the polyphenol content was significantly elevated under drought stress (18%) and normal conditions (15%), there was no significant difference in the polyphenol content under flood stress (Fig. 6).

## Discussion

PGPR are known to activate plant responses to counteract the adverse effects of biotic and abiotic stresses through plant growth-promoting metabolite production. This study elucidated the interaction of IAA producing *R. sphaeroides* with adzuki bean plants under flood stress and drought stress. .

In the current study, endogenous ABA content significantly increased in non-inoculated plants, which is a principal and typical response under abiotic stress [28], but upon inoculation of KE149, the endogenous plant ABA content was significantly reduced. The regulation of ABA in the current study was similar to our previous findings Shahzad *et al*. [20]. It is well known that JA and SA form a complex network that controls the defense mechanism against different types of microbes, eventually leading to plant resistance [29]. They interact both positively and negatively with major signaling pathways regulated by ethylene and JA to minimize oxidative stress in plants. In our study, the elevation of SA in KE149-inoculated plants indicated that plants might have experienced less oxidative stress [30]. Our results are in agreement with the findings of Sing and Usha [31] showing that SA application reduced stress and promoted wheat growth under water stress. Similar results were reported by Hayat *et al*. [32], who found that SA application mitigated water stress and promoted the growth of tomato plants. Similarly, JA has been reported to mediate several biotic and abiotic stresses; however, its role has been contradictory [33]. JA has been reported to improve resistance to chilling and drought in rice, tomato, and strawberry plants [33]. On the other hand, the exogenous application of methyl jasmonate could trigger lipid peroxidation, which is an indicator of stress-induced damage [34]. JA has also been reported to considerably decrease rice yield [35]. In the current study, JA was decreased in KE149-treated plants under stress, indicating that the reduction of JA may be attributed to the amelioration of the flood stress and drought stress of adzuki bean plants. A similar pattern of hormonal change was reported by Kang *et al*. [13]. The reduced level of ABA and JA and elevated level of SA may be associated with resistance in KE149-treated plants.

KE149 application significantly enhanced plant growth attributes such as the root and shoot fresh weight, root and shoot length, and chlorophyll content. An elevated chlorophyll content could increase the photosynthetic rate, which might enhance plant growth in a flood and drought environment. Our results are consistent with the findings of Kang *et al*. [15] demonstrating that *R. sphaeroides* KE149 inoculation greatly promoted the morphological attributes of cucumber plants through IAA. Since IAA has been reported to play a key role in promoting plant growth through nutrient assimilation from the soil, possible IAA synthesis by *R. sphaeroides* KE 149 in the root zone of plants might induce plant growth.

Existing evidence suggests that a high tolerance may be associated with osmotic balance. PGPR typically counteract toxic ion influx and maintain the osmotic balance in plants by regulating the ion transport system in plant tissues. Cations such as Na^+^, K^+^, and Ca^2+^ play an important role in the adaptation and sensitivity of plants [36]. A higher influx of Na^+^ may be considered as toxic to plants as it results in nutrient imbalance [37]. In the current study, KE149 inoculation reduced the uptake of Na^+^ and enhanced K^+^ uptake, which might maintain ion homeostasis in cells.

Osuagwu *et al*. [38] reported that water stress had no significant effect on the regulation of Mg and Ca in *Ocimum gratissimum*. Smith *et al*. [39] observed the significant loss of Ca and Mg in soybean plant tissues under drought. In our study, KE149-inoculated plants contained significantly higher levels of Mg^2+^ and Ca^2+^. Since IAA is reported for root rhizosphere engineering, possible IAA synthesis through KE149 might play a role in increasing the root surface area to acquire essential nutrients from the soil to alleviate stress.

Microorganisms have been reported to have several strategies to maintain osmotic balance through the accumulation of osmoprotectants containing important amino acids or their derivatives such as proline and glutamate[20]. A study has indicated that the accumulation of proline would promote high drought tolerance [40]. In the present study, methionine and proline were highly elevated during drought stress. Our results are in agreement with those of Agami *et al*. [41] who reported an increase in the proline content of basil plants exposed to water stress. A similar pattern of increase was observed for methionine in KE149-inoculated plants during flood stress. The elevation of methionine might have a possible effect on ethylene synthesis and oxygen availability in the root zone, thus conferring tolerance in plants. Most of the amino acids whose levels were increased by inoculated stressed plants are the precursors or intermediates of other metabolites important for abiotic stress resistance [42]. This demonstrates their role in further strengthening host plant defenses through different mechanisms, such as enhanced protein synthesis, increased growth rate under abiotic stresses [20].

Prolonged abiotic stress conditions may result in death due to the production of ROS [43]. The polyphenol content and DPPH radical scavenging activity are considered the major determinants of the quality of crops [44]. Polyphenols are derived from phenylpropanoids, which can enhance osmotic stress tolerance through toxic radicals and peroxides, protecting plants from the toxic effect of ROS [45]. Our results are consistent with those of Kang and Saltveit [46] showing that antioxidant enzymes and DPPH radical scavenging activity were increased in rice plants due to heat and chilling stress. Similar results were observed in our previous study Kang *et al*. [13] where DPPH radical scavenging activity was higher in soybean plants under salt and drought stress following inoculation with the rhizobacterium *P. putida*. Similarly, Nautiyal *et al*. [47] reported that *Bacillus lentimorbus* NRRL B-30488 application increased the total polyphenol content in vegetables. Furthermore, our results are in agreement with those of Rojas-Tapias *et al*. [48] showing that the polyphenol content was sharply increased under salt stress in maize leaves, and the polyphenol content was higher in *Azotobacter*-inoculated plants than in control plants. Antioxidant activity is dependent on hydrogen radicals dissociated from phenolic compounds that form a stable compound with DPPH radicals. The increased polyphenol content of KE149-inoculated plants under stress demonstrated the significance of PGPR in promoting antioxidant activities. Moreover, bacteria inoculation strengthen plants antioxidants system normalize the stressful condition which is also evident by less accumulation of stress responsive endogenous hormones (abscisic acid and jasmonic acid).

In conclusion, our results demonstrated the potential benefits of *R. sphaeroides* KE149 during water stress. The experimental data and evidence revealed that *R. sphaeroides* KE149 could tolerate water stress by synthesizing IAA and ABA, which could modulate endogenous phytohormones such as ABA, JA, and SA. The rhizobacterium *R. sphaeroides* KE149 was actively involved in the regulation of amino acids and antioxidants and played a key role in the acquisition of mineral elements, which ultimately promoted plant growth. This study sheds light on the potential and usefulness of phytohormone-producing rhizospheric bacteria such as *R. sphaeroides* KE149 for developing biologically safe bio-fertilizers that can enhance plant tolerance to water stress and thus improve the growth, development, and quality yield.

## Supplementary Data

The gene sequence of *R. sphaeroides* KE149 is available on the NCBI database with the accession number KY938536. The phylogenetic tree was constructed with the sequence obtained through 16S rRNA gene sequence analysis using MegaBLAST search program version 6 (Fig. S4).
Supplementary data for this paper are available on-line only at http://jmb.or.kr.

## Figures and Tables

**Table 1:** Effect of *R. sphaeroides* on the growth attributes of adzuki bean plants under water stress.

Treatment	Shoot length (cm/plant)	Root length (cm/plant)	Shoot weight (g/plant)	Root weight (g/plant)	Stem diameter (mm/plant)
NS	25.14 ± 1.38^b^	14.84 ± 0.21^b^	6.79 ± 0.12^b^	2.43 ± 0.05^d^	3.54 ± 0.10^b^
NSB	30.60 ± 0.71^a^	20.28 ± 0.55^a^	8.28 ± 0.25^a^	5.79 ± 0.11^a^	4.16 ± 0.10^a^
FS	24.76 ± 0.57^b^	13.34 ± 0.27^bc^	5.83 ± 0.13^c^	2.18 ± 0.15^e^	3.02 ± 0.04^c^
FSB	31.16 ± 0.93^a^	14.94 ± 0.15^b^	7.97 ± 0.12^a^	3.49 ± 0.11^b^	3.22 ± 0.18^bc^
DS	23.04 ± 0.26^b^	12.38 ± 0.28^c^	5.20 ± 0.15^d^	1.71 ± 0.04^f^	3.05 ± 0.06^c^
DSB	30.26 ± 0.63^a^	14.79 ± 0.30^b^	7.82 ± 0.11^a^	2.89 ± 0.11^c^	3.40 ± 0.21^b^

NS: No stress, NSB: No stress with bacteria, FS: Flood stress, FSB: Flood stress with bacteria, DS: Drought stress, DSB: Drought stress with bacteria. The superscript letters ^a^ and ^b^ after the mean values in a column indicate significant differences at *p* < 0.05. Each value represents the mean ± SD (*n* = 3).
