# Supplementary material for: Inoculation with Indole-3-Acetic Acid-Producing Rhizospheric Rhodobacter sphaeroides KE149 Augments Growth of Adzuki Bean Plants Under Water Stress
Source: J Microbiol Biotechnol. 2020 Feb 10;30(5):717–25. doi: 10.4014/jmb.1911.11063 (PMC9728276; doi:10.4014/jmb.1911.11063)
Supplement: Supplementary file 1 [file JMB-30-5-717-supple.pdf]

J. Microbiol. Biotechnol.
https://doi.org/10.4014/jmb.1911.11063

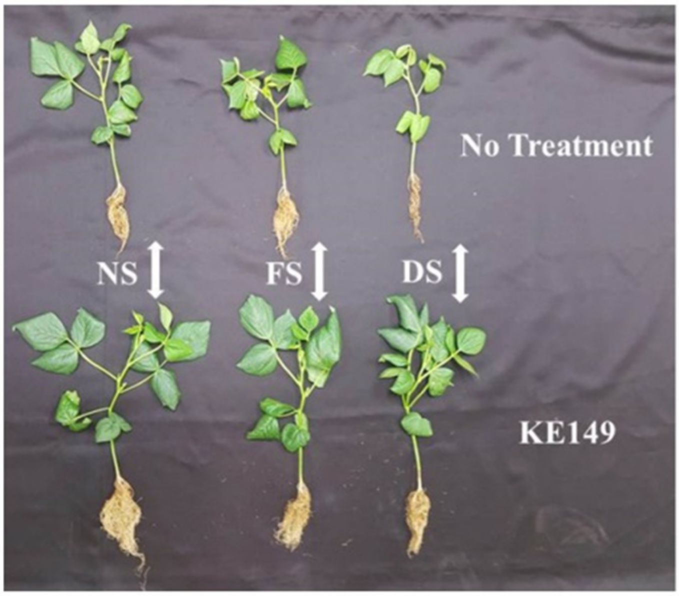

Supplementary Figure 1: Effect of *Rhodobacter sphaeroides* KE149 on morphological attributes of adzuki bean plant under flood and drought stress

Detection and quantification of Indole-3-acetic acid (IAA)

The bacterial broth culture was first centrifuged at 8160× *g* (Centrifuge, Eppendorf 5415C, Hamburg, Germany) for 10 min. The supernatant was separated and treated with 0.5 mL of The change in red color of KE149 inoculated broth was noted to confirm the strain ability to produce IAA. The method described by Lee et al. [1] was followed to quantify the IAA content in KE149 culture. In brief, 100mL of medium with 1 g/L of D-tryptophan was incubated at 30°C. The IAA was quantified after 7th and 10th day of incubation.

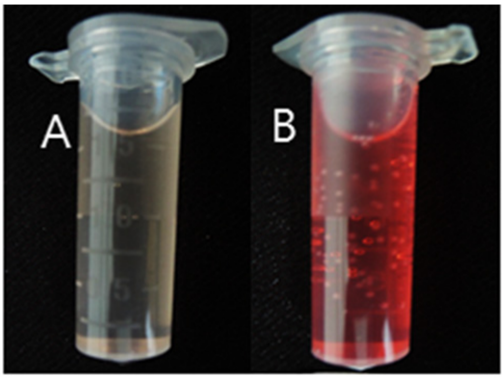

Supplementary Figure 2: Detection of Indole-3-acetic acid through Salkowski reagent test (A) Control (B) *Rhodobacter sphaeroides* KE149 culture.

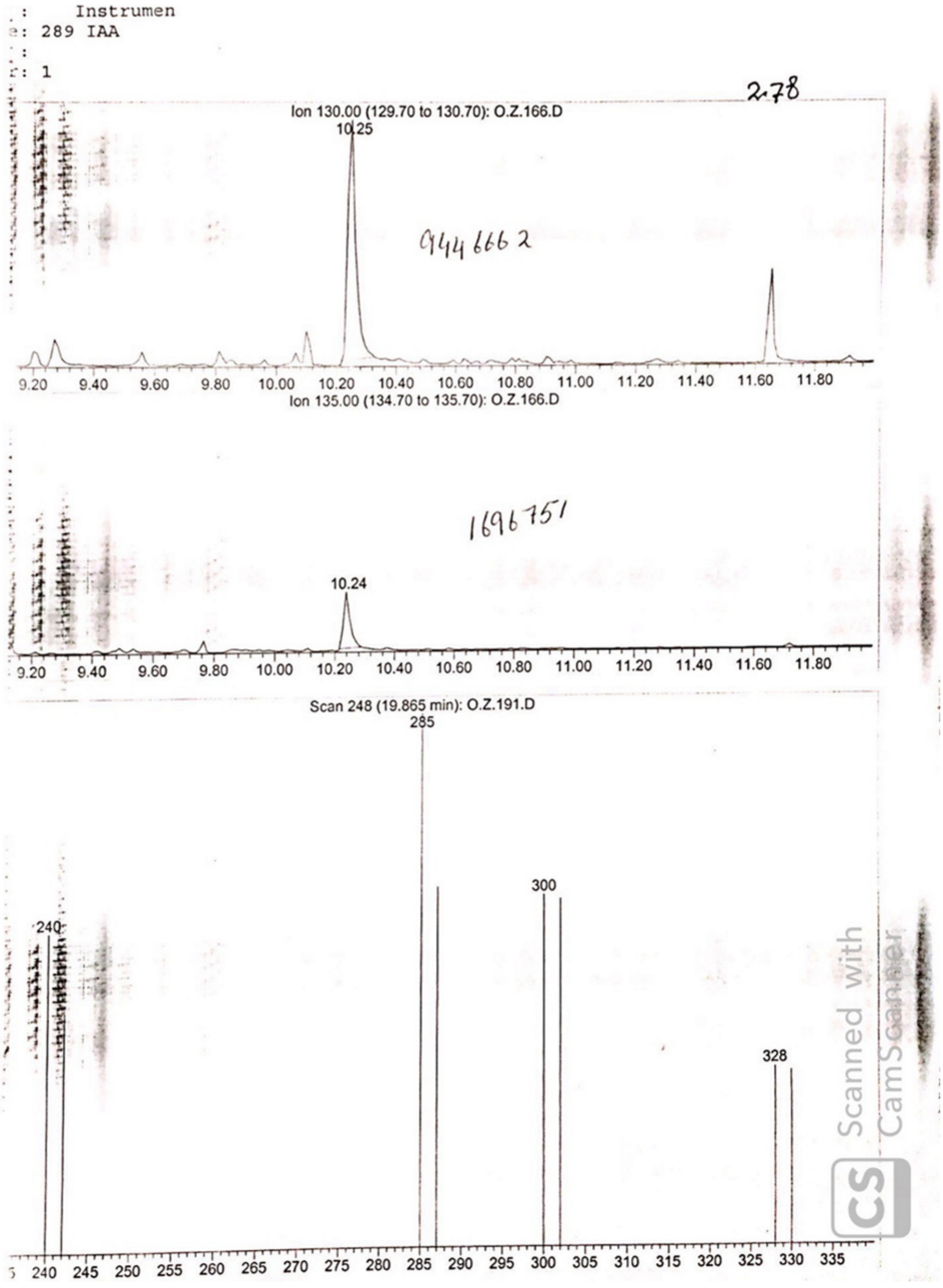

Supplementary Figure 3: GC–MS/SIM spectrometry analysis of IAA produced by *Rhodobacter sphaeroides* KE149

Supplementary Table. GC/MS – SIM conditions used for quantification of the indole-3-acetic acid

Equipment	Hewlett-Packard 6890, 5973N Mass Selective Detector
Column	HP-1 capillary column (30m×0.25mm i.d. 0.25μm film thickness) (J & W Scientific Co., Folsom, CA, USA)
Source temp.	230°C
Carrier gas	He (60 ml/min.); head pressure of 30 kPa
Oven conditions	70°C (2min.) → 20°C/min. → 280°C (5min)
Ionizing voltage	70 ev
Injector temp.	200°C

Molecular identification of bacterial isolate KE149

The 16S rRNA gene was amplified using a primer pair 27f (5'-AGA GTT TGA TCC TGG CTC AG-3') and 1492r (5'-TAC CTT GTT ACG ACT T-3'). The BLAST search program of NCBI GenBank database/EzTaxon was used to determine the nucleotide sequence homology of the targeted bacterial isolate. The highly related sequence with the highest homology and query coverage with lowest E-values were selected and aligned with clusatalW to construct phylogenetic tree.

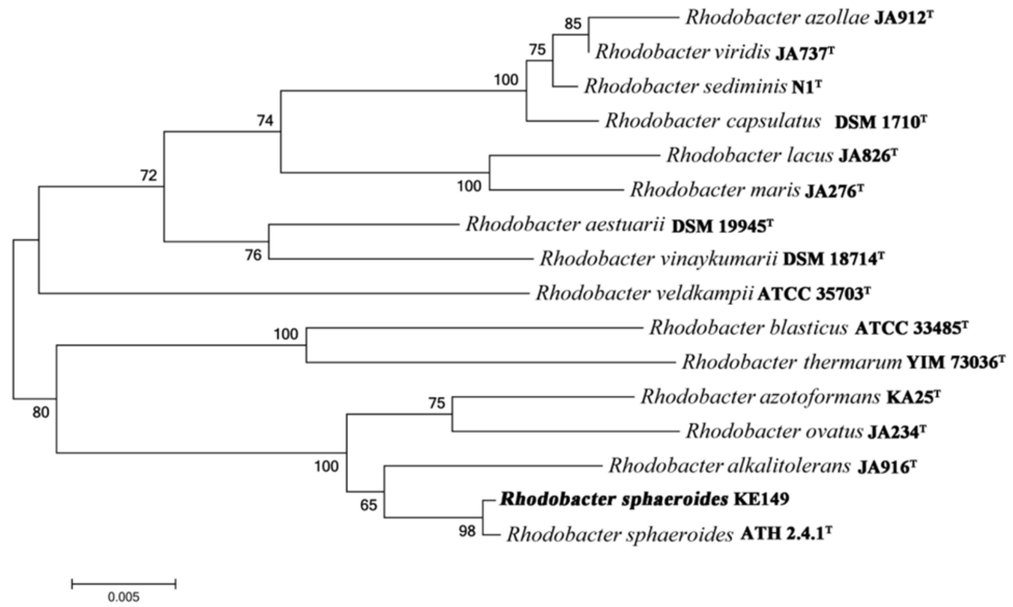

Supplementary Figure 4: Phylogentic relationships between 16S rRNA gene sequences from isolate KE149 and related bacterial srains. The sequence were aligned by CLUSTAL W and the tree was constructed by Neighbor Joining method using MEGAX software. Percentage confidence levels are indicated on each node that is generated from 500 boot strap.

References:

1. Lee, K.-E.; Radhakrishnan, R.; Kang, S.-M.; You, Y.-H.; Joo, G.-J.; Lee, I.-J.; Ko, J.-H.; Kim, J.-H., Enterococcus faecium LKE12 cell-free extract accelerates host plant growth via gibberellin and indole-3-acetic acid secretion. *J. Microbiol. Biotechnol* **2015,** 25, (9), 1467-1475.
